# UnMICST: Deep learning with real augmentation for robust segmentation of highly multiplexed images of human tissues

**DOI:** 10.1038/s42003-022-04076-3

**Published:** 2022-11-18

**Authors:** Clarence Yapp, Edward Novikov, Won-Dong Jang, Tuulia Vallius, Yu-An Chen, Marcelo Cicconet, Zoltan Maliga, Connor A. Jacobson, Donglai Wei, Sandro Santagata, Hanspeter Pfister, Peter K. Sorger

**Affiliations:** 1 Laboratory of Systems Pharmacology, Harvard Medical School, Boston, MA 02115 USA; 2 Image and Data Analysis Core, Harvard Medical School, Boston, MA 02115 USA; 3 School of Engineering and Applied Sciences, Harvard University, Cambridge, MA 02138 USA; 4 Ludwig Center for Cancer Research at Harvard, Harvard Medical School, Boston, MA 02115 USA; 5 Department of Pathology, Brigham and Women’s Hospital, Harvard Medical School, Boston, MA 02115 USA; 6 Department of Systems Biology, Harvard Medical School, Boston, MA 02115 USA

**Keywords:** Image processing, Cancer imaging, Machine learning

## Abstract

Upcoming technologies enable routine collection of highly multiplexed (20–60 channel), subcellular resolution images of mammalian tissues for research and diagnosis. Extracting single cell data from such images requires accurate image segmentation, a challenging problem commonly tackled with deep learning. In this paper, we report two findings that substantially improve image segmentation of tissues using a range of machine learning architectures. First, we unexpectedly find that the inclusion of intentionally defocused and saturated images in training data substantially improves subsequent image segmentation. Such real augmentation outperforms computational augmentation (Gaussian blurring). In addition, we find that it is practical to image the nuclear envelope in multiple tissues using an antibody cocktail thereby better identifying nuclear outlines and improving segmentation. The two approaches cumulatively and substantially improve segmentation on a wide range of tissue types. We speculate that the use of real augmentations will have applications in image processing outside of microscopy.

## Abstract (web summary)

Presenting UnMICST, strategies for robust single-cell segmentation in challenging human tissues.

## Introduction

The cell types, basement membranes, and connective structures that organize tissues and tumors are present on length scales ranging from subcellular organelles to whole organs (<0.1 to >10^4^ µm). Microscopy using Hematoxylin and Eosin (H&E) complemented by immunohistochemistry^1^ has long played a primary role in the study of tissue architecture^2,3^. Moreover, clinical histopathology remains the primary means by which diseases such as cancer are staged and managed clinically^4^. However, classical histology provides insufficient molecular information to precisely identify cell subtypes, study mechanisms of development, and characterize disease genes. High-plex imaging (Supplementary Table 1)^5–9^ of normal and diseased tissues (sometimes called spatial proteomics) yields subcellular resolution data on the abundance of 20–60 antigens, which is sufficient to identify cell types, measure cell states (quiescent, proliferating, dying, etc.) and interrogate cell signaling pathways. High-plex imaging also reveals the morphologies and positions of acellular structures essential for tissue integrity in a preserved 3D environment. High-plex imaging methods differ in resolution, field of view, and multiplicity (plex), but all generate 2D images of tissue sections; in current practice, these are usually 5–10 µm thick.

When multiplexed images are segmented and quantified, the resulting single cell data are a natural complement to single cell RNA Sequencing (scRNASeq) data, which have had a dramatic impact on our understanding of normal and diseased cells and tissues^10,11^. Unlike dissociative RNASeq, however, multiplex tissue imaging preserves morphology and spatial information. However, high-plex imaging data are substantially more challenging to analyze computationally than images of cultured cells, the primary emphasis of biology-focused machine vision systems to date. In particular, single cell analysis of imaging data requires segmentation, a computer vision technique that assigns class labels to an image in an instance or pixel-wise manner to subdivide it. The resulting segmentation mask is then used to quantify the intensities of different markers by integrating fluorescent signal intensities across each object (cell) identified by the mask or across a shape (usually an annulus) that outlines or is centered on the mask^12^. Extensive work has gone into the development of methods for segmenting metazoan cells grown in culture, but segmentation of tissue images is a more difficult challenge due to cell crowding and the diverse morphologies of different cell types. Recently, segmentation routines that use machine learning have become standard, paralleling the widespread use of convolutional neural networks (CNNs) in image recognition, object detection, and synthetic image generation^13^. Architectures such as ResNet, VGG16, and more recently, UNet and Mask R-CNN^14,15^ have gained widespread acceptance for their ability to learn millions of parameters and generalize across datasets, as evidenced by excellent performance in a wide range of segmentation competitions, as well as in hackathon challenges^16^ using publicly available image datasets^17,18^.

In both cultured cells and tissues, localizing nuclei is an optimal starting point for segmenting cells since most cell types have one nucleus (cells undergoing mitosis, muscle and liver cells and osteoclasts are important exceptions), and nuclear stains with high signal-to-background ratios are widely available. The nucleus is generally quite large (5–10 µm) relative to the resolution of wide-field fluorescence microscopes (~0.5 µm for a 0.9 numerical aperture – NA – objective lens), making it easy to detect at multiple magnifications. Nuclei are also often found at the approximate center of a cell. There are advantages to using additional markers during image acquisition; for example Schüffler et al.^19^ used multiplexed IMC data and watershed methods for multi-channel segmentation. However, it is not clear which proteins are sufficiently widely expressed in different cell types and tissues to be useful in segmentation. Methods based on random forests such as Ilastik and Weka^20,21^ exploit multiple channels for class-wise pixel classification via an ensemble of decision trees to assign pixel-wise class probabilities in an image. However, random forest models have far less capacity for learning than CNNs, which is a substantial disadvantage. Thus, the possibility of using CNNs with multi-channel data to enhance nuclei segmentation has not been widely explored.

A wide variety of metrics are used to quantify the performance of segmentation routines. These can be broadly divided into pixel and instance-level metrics; the former measures overlap in the shape and position of segmentation masks at the pixel level whereas the latter measures whether there is agreement in the presence or absence of a mask. The sweeping intersection over union (IoU; the Jaccard Index)^16^ is an example of a pixel-level performance metric; it is calculated by measuring the overlap between a mask derived from ground truth annotation and a predicted mask based on the ratio of the intersection of the pixels to their union. The greater the IoU, the higher the accuracy, with an ideal value of 1 (although this is very rarely achieved). The F1-score is an example of an instance-level metric that uses the weighted average of the precision (true positives normalized to predictions) and recall (true positives normalized to ground truth). A ‘positive’ in this case is commonly scored as 50% overlap (at the pixel level) between a predicted mask and the ground truth. It, therefore, accommodates substantial disagreement about the shape of the mask. In this context, it is important to note that supervised learning relies on the establishment of a ground truth by human experts. As described in detail below, for tissue imaging, the reported level of agreement among human experts for pixel-level annotation is only about 0.6 (at an IoU of 60%), suggesting that experts are themselves unable to determine the precise shape of segmentation masks (and the cells they represent). Not surprisingly, interobserver agreement is substantially higher (0.7–0.9) when evaluated using an instance level metric such as F1 score because it is relatively simple to decide whether a nucleus is present or not. As mentioned above, segmentation masks in high-plex imaging are commonly used to compute the integrated intensities of antibodies against nuclear, cytoplasmic, and cell-surface proteins and this places a premium on correctly determining the shape of the mask. Thus, the use of stringent pixel-level metrics such as IoU is essential for evaluating segmentation accuracy in single-cell analysis of multiplex tissue images.

The accuracy of segmentation by humans and computational methods is crucially dependent on the quality of the original images. In practice, many images of human and murine tissues have focus artefacts (blur) and images of some cells are saturated (with intensities above the linear range of the camera). This is particularly true of whole-slide imaging in which up to 1000 sequentially acquired image tiles are used to create mosaic images of specimens as large as several square centimeters. Whole slide imaging is a diagnostic necessity^22^ and essential to achieve sufficient power for rigorous spatial analysis^23^. However, many recent papers addressing the segmentation of tissue images restrict their analysis to the clearest in-focus fields. This is logical because, in the setting of supervised learning, it is easier to obtain training data and establish a ground-truth when images are clear and inter-observer agreement is high. In practice, however, all microscopy images of tissue specimens have issues with focus: the depth of field of objective lenses capable of high resolution imaging (high NA lenses) is typically less than the thickness of the specimen so that objects above and below the plane of optimal focus are blurred. Images of human biopsy specimens are particularly subject to blur and saturation artefacts because the tissue sections are not always uniformly co-planar with the cover slip. Since most research on human tissues is incidental to diagnosis or treatment, it is rarely possible to reject problematic specimens outright. Moreover, reimaging of previously analyzed tissue sections is rarely possible due to tissue disintegration. Thus, image segmentation with real-world data must compensate for common image aberrations.

The most common way to expand training data to account for image artefacts is via computational augmentation^24^ which involves pre-processing images via random rotation, shearing, flipping, etc. This is designed to prevent algorithms from learning irrelevant aspects of an image, such as orientation. To date, focus artefacts have been tackled using computed Gaussian blur to augment training data^25–27^. However, Gaussian blur is only an approximation of the blurring inherent to any optical imaging system having limited bandpass (that is—any real microscope) plus the effects of refractive index mismatches and light scattering.

In this paper, we investigate ways to maximize the accuracy of image segmentation by machine learning algorithms in multiplexed tissue images containing common imaging artefacts. We generate a set of training and test data with ground-truth annotations via human curation of multiple normal tissues and tumors, and use these data to score segmentation accuracy achieved on three deep learning networks, each of which was independently trained and evaluated: UNet, Mask R-CNN, and Pyramid Scene Parsing Network (PSPNet). The resulting models comprise a family of *Universal Models for Identifying Cells and Segmenting Tissue* (UnMICST) in which each model is based on the same training data but a different class of ML network. Based on our analysis we identify two ways to improve segmentation accuracy for all three networks. The first involves adding images of nuclear envelope staining (NES) to images of nuclear chromatin acquired using DNA-intercalating dyes. The second involves adding real augmentations, defined here as intentionally defocused and over-saturated images (collected from the same specimens), to the training data to make models more robust to the types of artefacts encountered in real tissue images. We find that augmentation with real data significantly outperforms conventional Gaussian blur augmentation, offering a statistically significant improvement in model robustness. Across a range of tissue types, improvements from adding NES data and real augmentations are cumulative.

## Results

### Data sets and ground truth annotation of nuclear boundaries

One challenge in supervised machine learning on tissue images is a lack of sufficient freely-available data with ground truth labeling. Experience with natural scene images^14^ has shown that the acquisition of labels can be time consuming and rate limiting^28^. It is also well established that cells in different types of tissue have nuclear morphologies that vary substantially from the spherical and ellipsoidal shape observed in cultured cells^29^. Nuclear pleomorphism (variation in nuclear size and shape) is even used in histopathology to grade cancers^30^. To account for variation in nuclear morphology we generated training, validation, and test datasets from seven different tissue and tumor types (lung adenocarcinoma, non-neoplastic small intestine, normal prostate, colon adenocarcinoma, glioblastoma, non-neoplastic ovary, and tonsil) found in 12 cores from EMIT (Exemplar Microscopy Images of Tissue^31^, RRID: SCR_021052), a tissue microarray assembled from clinical discards. The tissues had cells with nuclear morphologies ranging from mixtures of cells that were large vs. small, round cells vs. narrow, and densely and irregularly packed vs. organized in clusters. A total of ~10,400 nuclei were labeled by a human expert for nuclear contours, centers, and background. In addition, two human experts labeled a second dataset from a whole-slide image of human melanoma^32^ to establish the level of inter-observer agreement and to provide a test data set that was disjoint from the training data.

### Evaluating the performance of ML segmentation algorithms and models

We implemented and then evaluated two semantic and one instance segmentation algorithms that are based on deep learning/CNNs (UNet, PSPNet, and Mask R-CNN, respectively). Semantic segmentation is a coarse-grained ML approach that assigns objects to distinct trained classes, while instance segmentation is fine grained and identifies individual instances of objects. We trained each of these models (UnMICST-U, UnMICST-P, and UnMICST-M, respectively) on manually curated and labeled data from seven distinct tissue types. The models were not combined, but were tested independently in an attempt to determine which network exhibited the best performance.

We evaluated performance using both pixel- and instance-level metrics including the sweeping intersection over union (IoU) threshold described by Caicedo et al.^16^, which is based on images of cell lines, and implemented in the widely used COCO dataset^33^. The IoU (the Jaccard Index) is calculated by measuring the overlap between the ground truth annotation and the prediction via a ratio of the intersection to the union of pixels in two masks. The (IoU) threshold is evaluated over a range of values from the least stringent, 0.55, to most stringent, 0.8^16^. Unlike a standard pixel accuracy metric (the fraction of pixels in an image that were correctly classified), IoU is not sensitive to class-imbalance. IoU is a particularly relevant measure of segmentation performance for analysis of high-plex images. When masks are used to quantify marker intensities in other channels, we are concerned not only with whether a nucleus is present or not at a particular location but whether the masks are the correct size and shape.

Examples of instance-level metrics are true positives (TP) and true negatives (TN), which classify predicted objects based on whether they overlap by 50% or greater, otherwise they are deemed as false positives (FP) and false negatives (FN). The frequencies of these four states are used to calculate the F1-score and average precision (AP). The F1-score is the weighted average of precision (true positives normalized to predictions) and recall (true positives normalized to ground truth), and AP considers the number of true positives, total number of ground truth, and predictions.

The accuracy expected for these methods was determined by having multiple human experts label the same set of data and determine the level of inter-observer agreement. We assessed inter-observer agreement using both the F1-score and sweeping IoU scores with data from whole-side images of human melanoma^32^. For a set of ~4900 independently annotated nuclear boundaries, two experienced microscopists achieved a mean F1-score of 0.78 (Supplementary Information 1) and an IoU of 60% at a threshold of 0.6. In the discussion, we compare these data to values obtained in other recently published papers and address the discrepancy in F1-scores and IoU values. We also discuss how these values might be increased to achieve super-human performance^24,34^.

### Real augmentations increase model robustness to focus artefacts

**Fig. 1:**
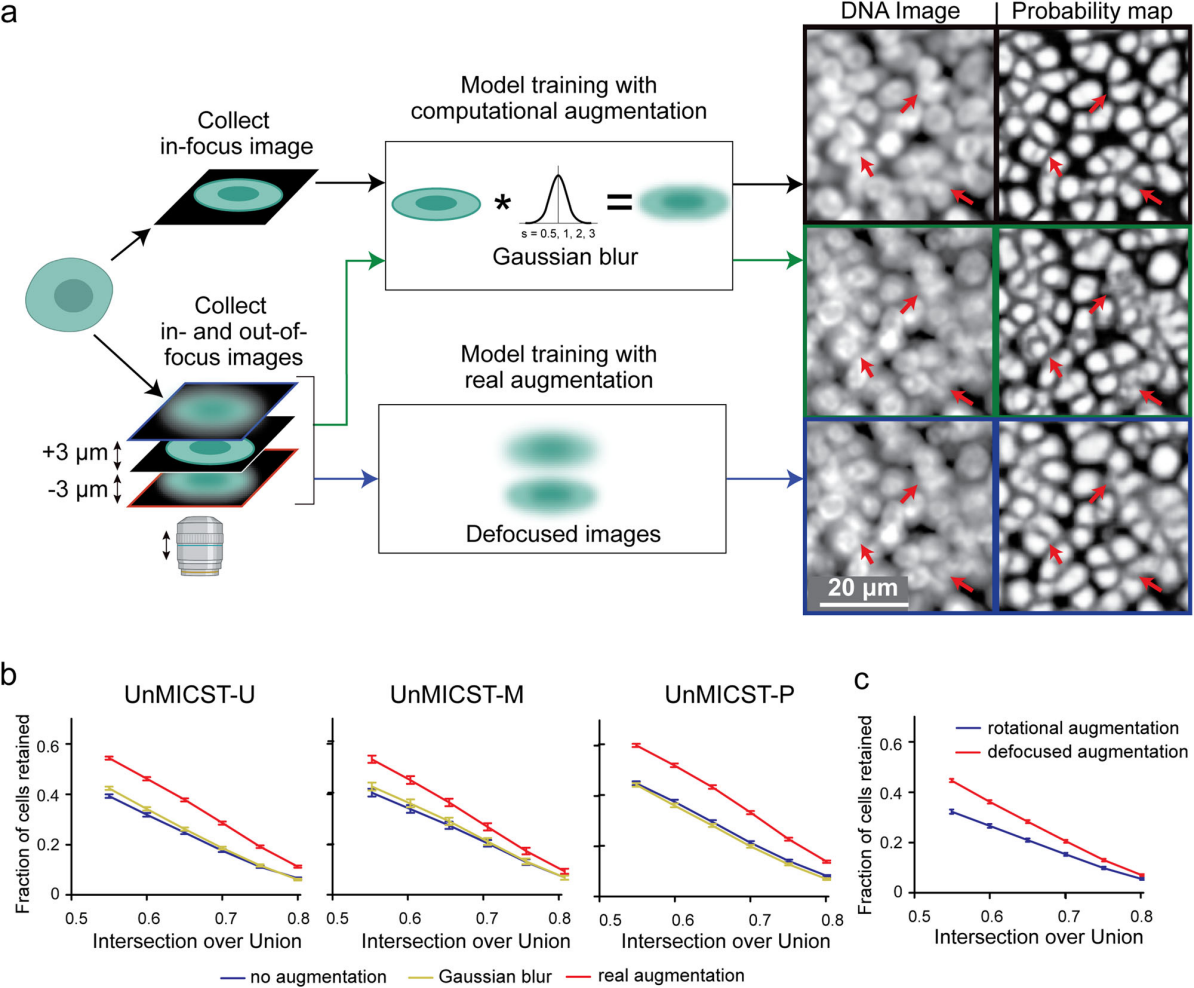
Comparing the use of real augmentations (defocused and overexposed images) and Gaussian blur. **a** Schematic diagram showing the approach comparing test images on models trained with Gaussian-blurred or defocused image data. Higher contrast probability maps signify more confidence— areas of interest are highlighted with red arrows. Corresponding probability maps indicate a model trained with defocused images performs better on defocused test images than a Gaussian-blurred model. Scale bar denotes 20 μm. **b** Plots show that incorporating real augmentations (red curve) into the training set is statistically significantly superior to training sets with Gaussian blur (yellow curve) and without real augmentations (blue curve) for UnMICST-U, UnMICST-M, and UnMICST-P. Simulating defocused images with Gaussian blur is only marginally better than not augmenting the training data at all. **c** Comparing UnMICST-U model accuracy when the training dataset size was held constant by replacing defocused augmentations (red curve) with 90 and 180° rotations (blue curve). Error bars are standard error of mean.

To study the impact of real and computed augmentations on the performance of segmentation methods, we trained models with different sets of data involving both real and computed augmentations and then tested the data on images that were acquired in focus, out of focus or blurred using a Gaussian kernel. Where dataset sizes were unbalanced, we supplemented such instances with rotation augmentations. We assessed segmentation accuracy quantitatively based on IoU and qualitatively by visual inspection of predicted masks overlaid on image data. Real augmentation involved adding additional empirical, rather than computed, training data having the types of imperfections most commonly encountered in tissue. This was accomplished by positioning the focal plane 3 µm above and below the specimen, resulting in de-focused images. A second set of images was collected at long exposure times, thereby saturating 70–80% of pixels. Because blurred and saturated images were collected sequentially without changing stage positions, it was possible to use the same set of ground truth annotations. For computed augmentations, we convolved a Gaussian kernel with the in-focus images using a range of standard deviations chosen to cover a broad spectrum of experimental cases (Fig. 1a). In both scenarios, the resulting models were evaluated on a test set prepared in the same way as the training set.

In an initial set of studies, we found that models created using training data augmented with Gaussian blur performed well on Gaussian blurred test data. However, when evaluated against test data involving defocused and saturated images, we found that Gaussian blur augmentation improved accuracy only slightly relative to baseline models lacking augmentations (Fig. 1b). In contrast, the use of training data supplemented with real augmentations increased the fraction of cells retained at an IoU threshold of 0.6 by 40–60%. Statistically significant improvement was observed up to an IoU cutoff of 0.8 with all three learning frameworks (UnMICST-U, UnMICST-M, and UnMICST-P models). To perform a balanced comparison, we created two sets of training data having equal numbers of images. The first set contained the original data plus computed 90- and 180° rotations, and the second set contained original data plus defocused data collected from above and below the specimen. Again, we found that models trained with real augmentations substantially outperformed rotationally augmented models when tested on defocused test data (Fig. 1c). Thus, training any of the three different deep learning architectures with real augmentation generated models that outperformed models with computed augmentation using test data that contained commonly encountered artefacts.

### Addition of NES improves segmentation accuracy

**Fig. 2:**
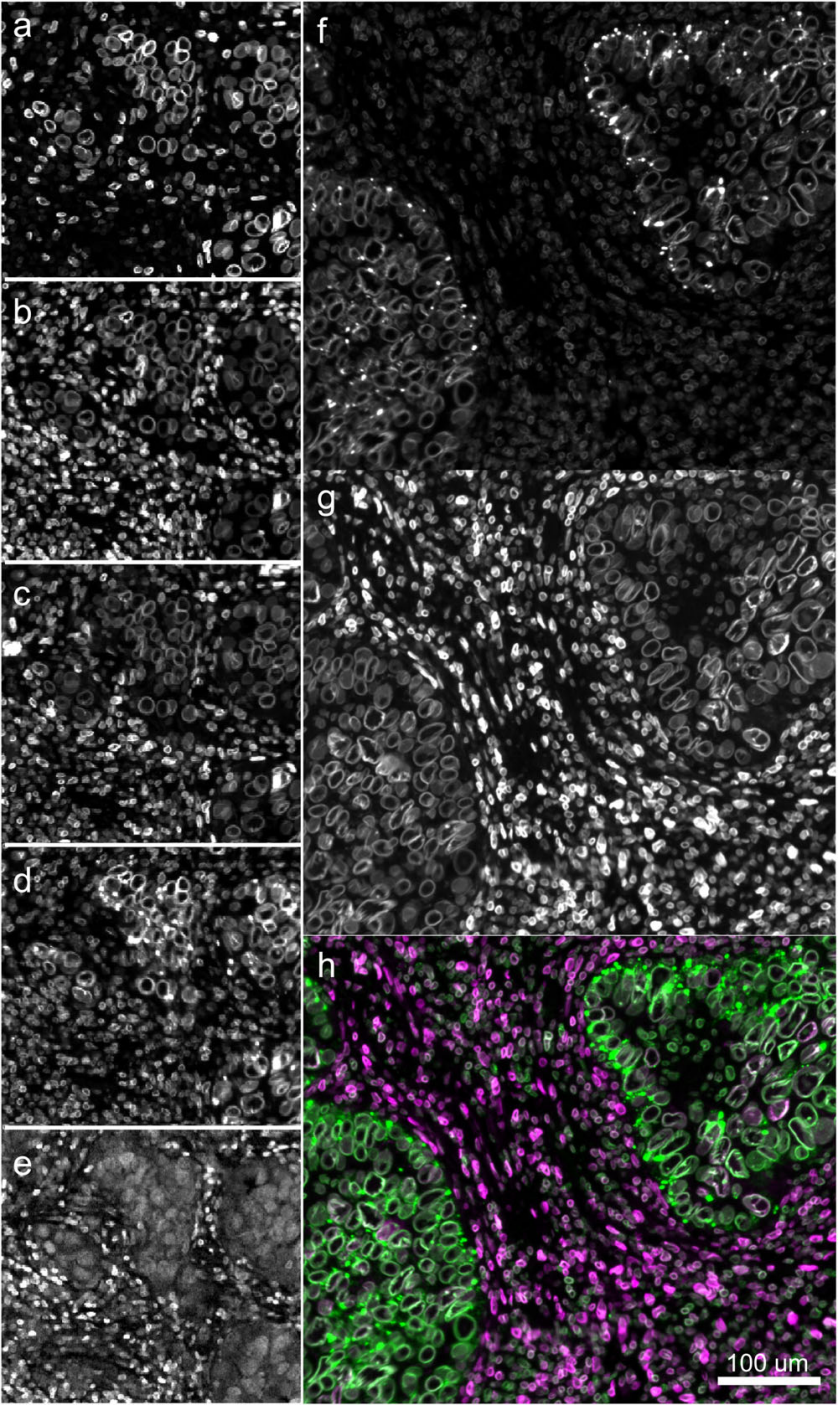
Comparing different nuclear envelope stains in colon adenocarcinoma. Showcasing **a** lamin A/C, **b** lamin B1, **c** lamin B2, **d** NUP98, and **e** the lamin B receptor in the same field of view. Lamin B1 and B2 appear to stain similar proportions of nuclei while lamin A/C stains fewer nuclei. The stain against the lamin B receptor was comparatively weaker. Lamin B2 (**f**) and NUP98 (**g**) are complementary and, when used in combination, maximize the number of cells stained. **h** Composite of lamin B2 (purple) and NUP98 (green). Scale bar denotes 100 μm.

When we stained our TMA panel (the Exemplar Microscopy Images of Tissues and Tumors (EMIT) TMA) we found that antibodies against lamin A and C (Fig. 2a) (which are different splice forms of *LMNA* gene) stained approximately only half as many nuclei as antibodies against lamin B1 (Fig. 2b) or lamin B2 (Fig. 2c) (products of the *LMNB1* and *LMNB2* genes). Staining for the lamin B receptor (Fig. 2e) exhibited poor image contrast. A pan-tissue survey showed that a mixture of antibodies for nucleoporin NUP98 (Fig. 2d) and lamin B2 conjugated to the same fluorophore (Alexafluor-647) generated nuclear envelope staining (NES) for nearly all nuclei across multiple tissues (Fig. 2f–h). We judged this to be the optimal antibody cocktail. However, only some cell types, epithelia in colorectal adenocarcinoma for example, exhibited the ring-like structure that is characteristic of nuclear lamina in cultured epithelial cells. The nuclear envelope in immune and other cells has folds and invaginations^35^ and in our data, NES staining could be irregular and diffuse, further emphasizing the difficulty of finding a broadly useful NES stain in tissue.

**Fig. 3:**
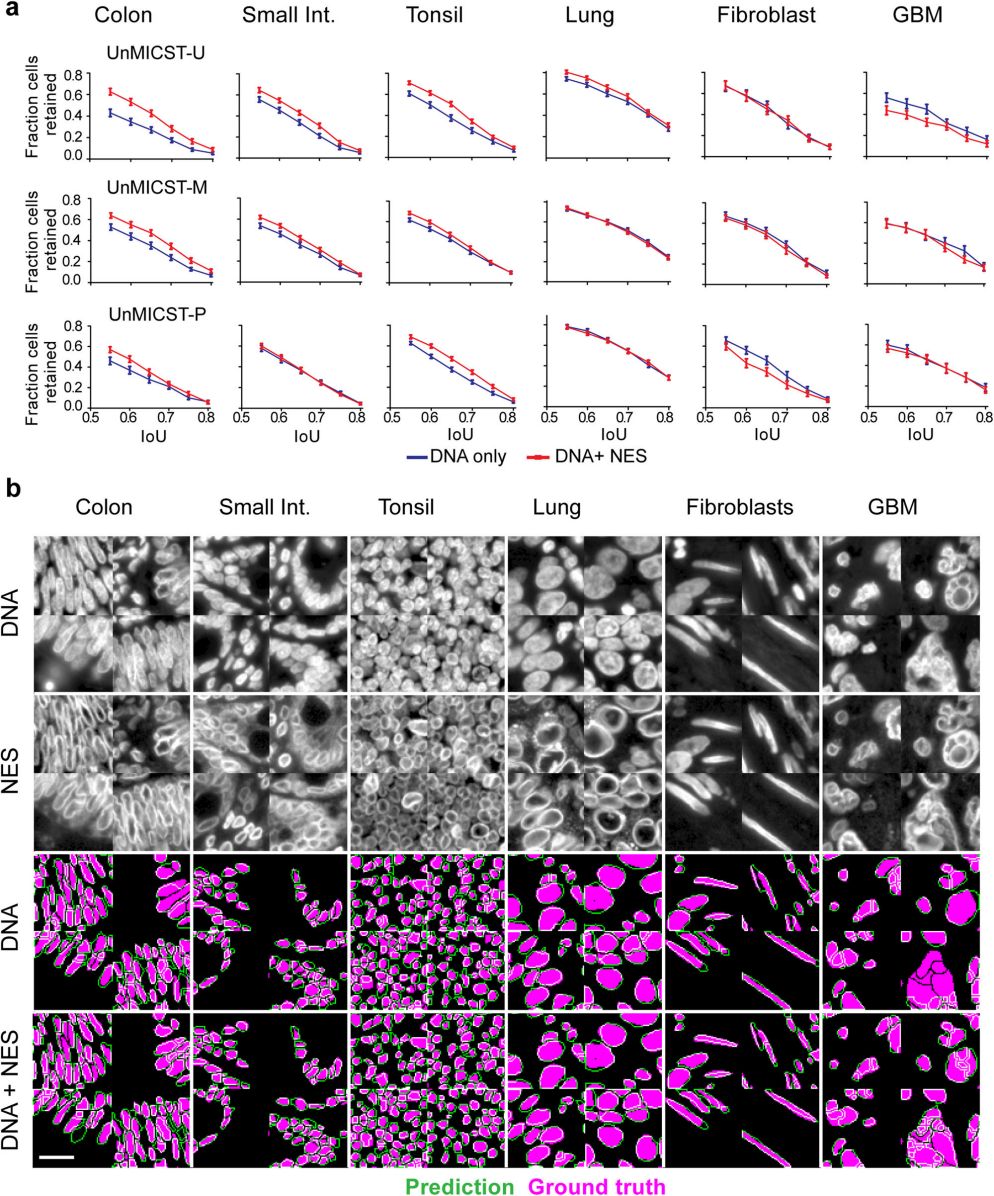
NES with DNA improves nuclear segmentation. NES – nuclear envelope staining. Assessing the addition of NES as a 2nd marker to DNA on segmentation accuracy on a per tissue and per model basis. **a** Variable IoU plots comparing the DNA-only model (blue curve) and the DNA + NES model (red curve) across frameworks. Adding NES increased accuracy for densely packed nuclei such as colon, small intestine, tonsil, and to some extent, lung tissue. Error bars are standard errors of mean. **b** Representative grayscale images of tissues stained with DNA and NES comparing their variable morphologies, followed by UnMICST-U mask predictions (green) overlaid onto ground truth annotations (purple). In tissue with sparse nuclei, such as fibroblasts from prostate tissue, NES did not add an additional benefit to DNA alone. In tissues where NES does not exhibit the characteristic nuclear ring, as in glioblastoma, the accuracy was similarly not improved. Scale bar denotes 20 μm.

The value of NES images for model performance was assessed quantitatively and qualitatively. In images of colon adenocarcinoma, non-neoplastic small intestine, and tonsil tissue, we found that the addition of NES images resulted in significant improvements in segmentation accuracy based on IoU with all three learning frameworks; improvements in other tissues, such as lung adenocarcinoma, were more modest and sporadic (Fig. 3a, Lung). For nuclear segmentation of fibroblasts in prostate cancer tissue, UnMICST-U and UnMICST-M models with NES data were no better than models trained on DNA staining alone. Most striking were cases in which NES data slightly decreased performance (UnMICST-P segmentation on prostrate fibroblasts and UnMICST-U segmentation of glioblastoma). Inspection of the UnMICST-P masks suggested that the segmentation of well-separated fibroblast nuclei was already optimal with DNA images alone (~60% of nuclei retained at IoU of 0.6), implying that the addition of NES images afforded little improvement. With UnMICST-U masks in glioblastoma, the problem appeared to involve atypical NES morphology, which is consistent with a high level of nuclear pleomorphism and the presence of giant cells, both of which are well-established features of high-grade glioblastoma^36,37^. We also note that NES data alone was inferior to DNA staining as a sole source of training data and should therefore be used in combination with images of DNA (Supplementary Information 2). Thus, adding NES to training data broadly but not universally improves segmentation accuracy.

### Combining NES images and real augmentation has a cumulative effect

**Fig. 4:**
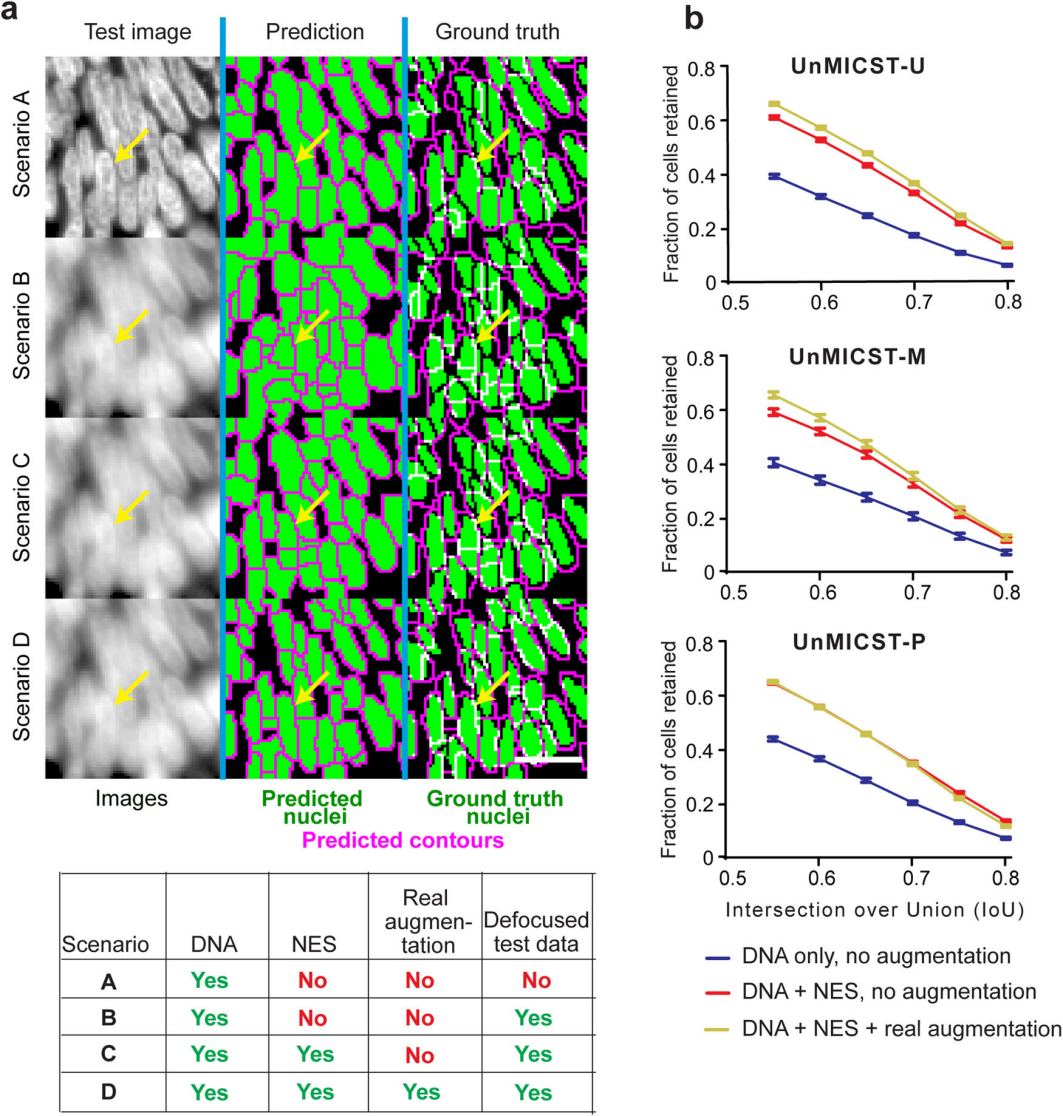
Combination of NES and real image augmentations on segmentation performance. NES - nuclear envelope staining. **a** Models trained with in-focus DNA data alone produced probability maps that were undersegmented, especially in densely-packed tissue such as small intestine (Scenario A). When tested on defocused data, nuclei borders were largely incorrect (Scenario B). Adding NES restored nuclei border shapes (Scenario C). Combining NES and real augmentations reduced false positive detections and produced nuclei masks better resembling the ground truth labels (Scenario D). Scalebar denotes 20 μm. Table legend shows conditions used for each scenarios A–D. Yellow arrow indicates a blurry cell of interest where accuracy improves with NES and real augmentation. **b** Graphs compare the accuracy represented as the number of cells retained across varying IoU thresholds with all models from UnMICST-U (top), UnMICST-M (center), and UnMICST-P (bottom). In all models, more nuclei were retained when NES and real augmentations were used together during training (yellow curves) compared to using NES without real augmentations (red curves) or DNA alone (blue curves). Error bars are standard error of mean.

To determine whether real augmentation and NES would combine during model training to achieve superior segmentation precision relative to the use of either type of data alone, we trained and tested models under four different scenarios (using all three learning frameworks; Fig. 4). We used images from the small intestine, a tissue containing nuclei having a wide variety of morphologies, and then extended the analysis to other tissue types (see below). Models were evaluated on defocused DNA test data to increase the sensitivity of the experiment. In the first scenario, we trained baseline models using in-focus DNA image data and tested models on unseen in-focus DNA images. With tissues such as the small intestine, which are challenging to segment because they contain densely-packed nuclei, scenario A resulted in slightly under-segmented predictions. In Scenario B and for all subsequent scenarios, defocused DNA images were included in the test set, giving rise to contours that were substantially misaligned with ground truth annotations and resulted in higher undersegmentation. False-positive predictions and imprecise localizations of the nuclei membrane were observed in areas devoid of nuclei and with very low contrast (Fig. 4a). When NES images were included in the training set (Scenario C), nuclear boundaries were more consistent with ground truth annotations, although false-positive predicted nuclei still remained. The most robust performance across ML frameworks and tissues was observed when NES images and real augmentation were combined: accurate nuclear boundaries were generally well aligned with ground truth annotations in both shape and in size. Observable differences in the placement of segmentation masks were reflected in improvements in IoU: for all three deep learning frameworks, including NES data and real augmentations increased the fraction of nuclei retained by 50% at an IoU threshold of 0.6 (Fig. 4b). The accuracy of UnMICST-P (blue curve) trained on in-focus DNA data alone was higher than the other two baseline models at all IoU thresholds, suggesting that UnMICST-P has a greater capacity to learn. UnMICST-P may have an advantage in experiments in which staining the nuclear envelope proves difficult or impossible.

### Combining NES and real augmentation is advantageous across multiple tissue types

**Fig. 5:**
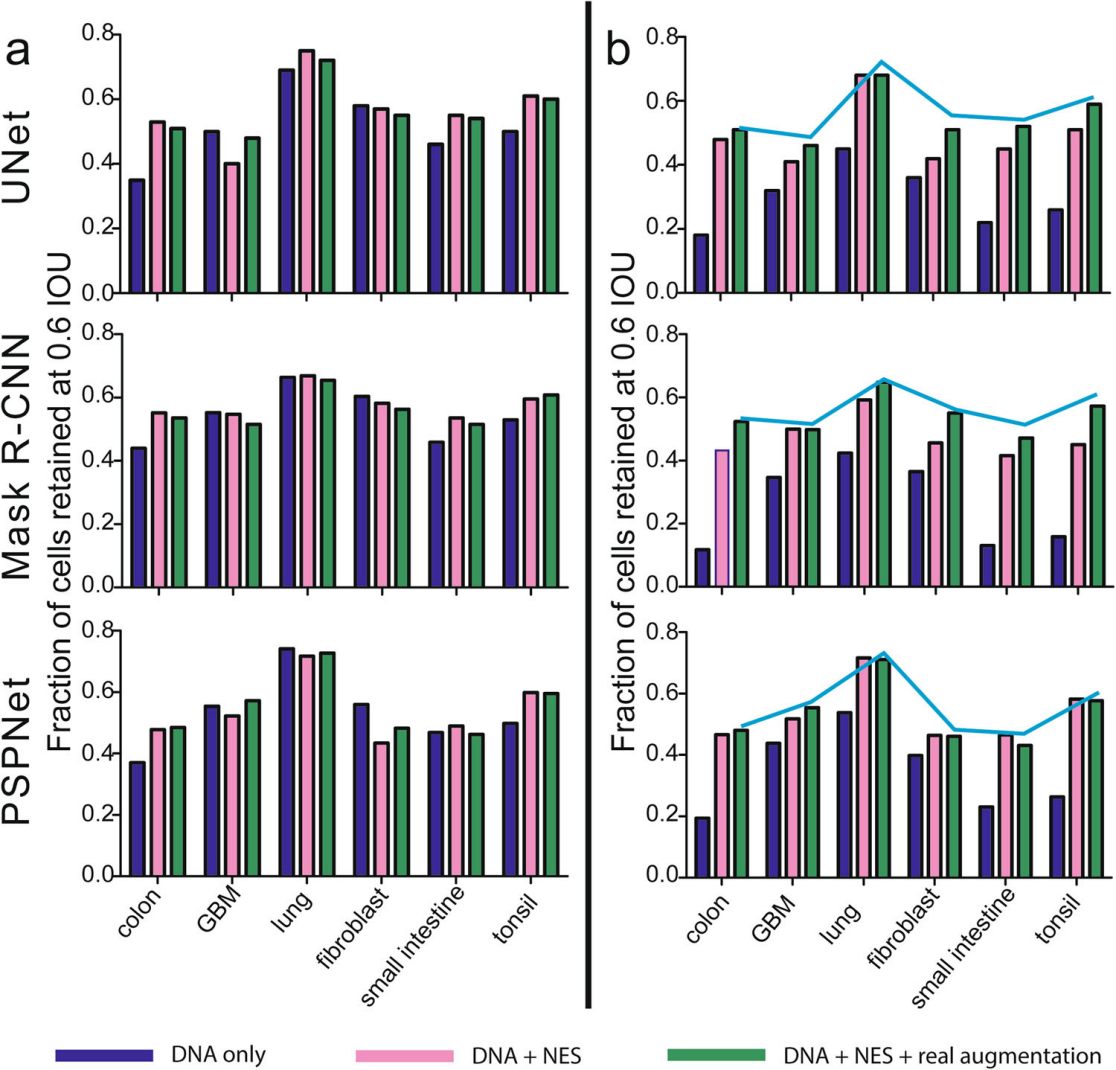
Assessing different training strategies on (a) in-focus and (b) defocused test data for different tissue types. **a** In all tissue types apart from GBM, the addition of NES (pink bars) and the use of real augmentations combined with NES (green bars) in training data offered superior accuracy compared to using DNA alone (blue bars). **b** When the models were tested on defocused data, all tissues (including GBM unexpectedly) showed benefits resulting from using NES (pink bars) combined with real augmentations (green bars). The line plot indicates highest accuracy achieved for each tissue when tested on in-focus data from panel (**a**).

To determine if improvements in segmentation would extend to multiple tissue types we repeated the analysis described above using three scenarios for training and testing with both in-focus (Fig. 5a) and defocused images (Fig. 5b). Scenario 1 used in-focus DNA images for training (blue bars), scenario 2 used in-focus DNA and NES images (red bars), and scenario 3 used in-focus DNA and NES images plus real augmentation (green bars). While the magnitude of the improvement varied with tissue type and test set (panel a vs b), the results as a whole support the conclusion that including both NES and real augmentations during model training confers statistically significant improvement in segmentation accuracy with multiple tissue types and models. The accuracy boost was greatest when models performed poorly (e.g., in scenario 1 where models were tested on defocused colon image data; Fig. 5b, blue bars), so that segmentation accuracy became relatively uniform across tissue and cell types. As a final test, we re-examined the whole slide melanoma image described above (which had not been included in any training data) and evaluated IoU, AP, and F1-scores. The data were consistent regardless of metric and showed that all three models benefitted from the inclusion of training data that included NES images and real augmentations (Supplementary Information 3). The improvement in accuracy, however, was modest and similar to lung adenocarcinoma. We attribute this to the fact that, like lung adenocarcinoma, melanoma has less dense regions, which our baseline models already performed well on.

### Applying UnMICST to highly multiplex whole-slide tissue images

**Fig. 6:**
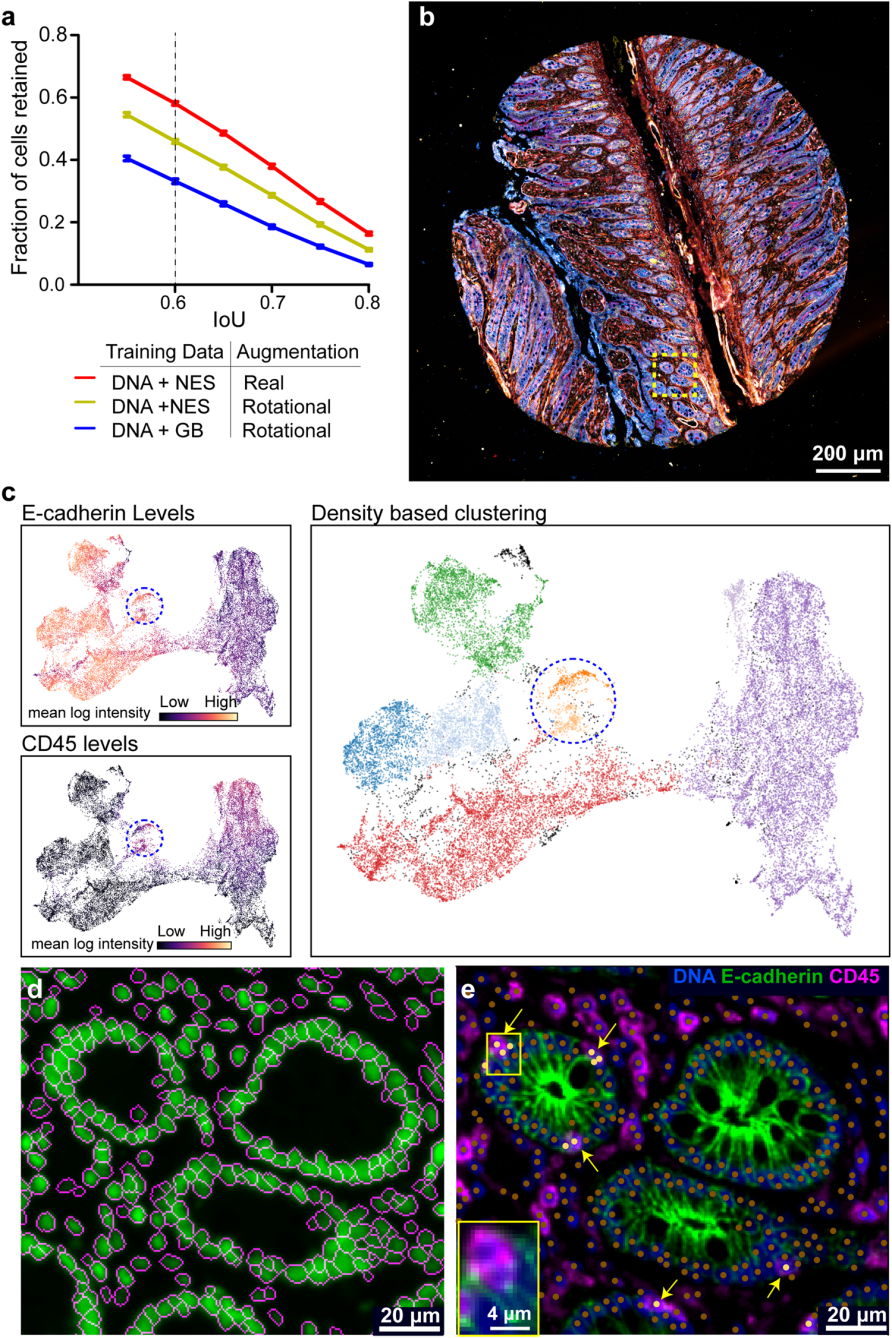
Applying UnMICST models to highly multiplexed image data. **a** Accuracy improvement of UnMICST-U models trained with and without NES (nuclear envelope staining) as compared to DNA alone, and real augmentations as compared to computed blur (GB; Gaussian blur). To balance training dataset size, GB was substituted for NES data and computed 90/180° rotations were substituted for real augmentations. Error bars are standard error of mean. **b** A 64-plex CyCIF image of a non-neoplastic small intestine TMA core from the EMIT dataset. Dashed box indicates region of interest for panels (**d**, **e**). **c** UMAP projection using single cell staining intensities for 14 marker proteins (see methods). The color of the data points represents the intensity of E-cadherin (top left) or CD45 (bottom left) across all segmented nuclei. Density-based clustering using HDBSCAN identified distinct clusters (each denoted by a different color) that were positive for either E-cadherin or CD45 as well as a small number of double-positive cells (blue dashed circle). **d** Enlarged region of yellow dashed box from **b** showing segmentation mask outlines (magenta) overlayed onto DNA channel (green). **e** Composite image of DNA, E-cadherin, and CD45 of the same region. Nuclei centroids from segmentation denoted by brown dots. Cells positive for both E-cadherin and CD45 (from blue dashed circle in panel **c** are marked with yellow arrows and yellow dots. Inset: enlarged view of boxed region showing overlapping immune and epithelial cells.

To investigate the overall improvement achievable with a representative UnMICST model, we tested UnMICST-U with and without real or computed augmentations and NES data on all six tissues as a set, including in-focus, saturated, and out-focus images (balancing the total amount of training data in each case). A 1.7-fold improvement in accuracy was observed at an IoU of 0.6 for the fully trained model (i.e., with NES data and real augmentations; Fig. 6a). Inspection of segmentation masks also demonstrated more accurate contours for nuclei across a wide range of shapes. The overall improvement in accuracy was substantially greater than any difference observed between semantic and instance segmentation frameworks. We, therefore, focused subsequent work on the most widely used framework: U-Net.

We also tested a fully trained UnMICST-U model on a 64-plex CyCIF image of non-neoplastic small intestine tissue from the EMIT TMA (Fig. 6b). Staining intensities were quantified on a per-cell basis, and the results visualized using Uniform Manifold Approximation and Projection (UMAP; Fig. 6c). Segmentation masks were found to be well-located with little evidence of under or over-segmentation (Fig. 6d). Moreover, whereas 21% of cells with segmented nuclei stained positive (as determined by using a Gaussian-mixture model) for the immune cell marker CD45, and 53% stained positive for the epithelial cell marker E-cadherin, less than 3% were positive for both. No known cell type is actually positive for both CD45 and E-cadherin, and the very low abundance of these double-positive cells is evidence of accurate segmentation. When we examined some of the 830 double positive cells (blue dashed circle in Fig. 6c) we found multiple examples of a CD3^+^ T cell (yellow arrowheads; light yellow dots in Fig. 6e) tightly associated with or between the epithelial cells of intestinal villi (green kiwi-like structure visible in Fig. 6e). This is consistent with the known role of the intestinal epithelium in immune homeostasis^38^. In these cases, the ability of humans to distinguish immune and epithelial cells relies on prior knowledge, multi-dimensional intensity features and subtle differences in shape and texture—none of which were aspects of model training. Thus, future improvements in tissue segmentation are likely to require the development of CNNs able to classify rare but biologically interesting spatial arrangements, rather than simple extensions of the general purpose segmentation algorithms described here.

### Some tissues still pose a challenge for nuclei segmentation

Of all the tissue types annotated and tested in this paper, non-neoplastic ovary was the most difficult to segment (Supplementary Information 4a) and addition of ovarian training data to models trained on data from other tissues decreased overall accuracy (Supplementary Information 4b). We have previously imaged ovarian cancers at even higher resolution (60×/1.42NA sampled at 108 nm pixel size)^39^ using optical sectioning and deconvolution microscopy; inspection of these images reveals nuclei with highly irregular morphology, poor image contrast, and dense packing (Supplementary Information 4c) unlike colon adenocarcinoma (Supplementary Information 4d). Thus, additional research, possibly involving different NES antibodies, will be required to improve performance with ovarian and other difficult to segment tissues. Until then, caution is warranted when combining training data from tissues with very different nuclear morphologies.

## Discussion

This paper makes four primary contributions to the growing literature on the segmentation of tissue images, which is an essential step in single-cell data analysis. First, it explicitly considers training and test data that contain the types of focus and intensity artefacts that are commonly encountered in whole-slide images, particularly images of human tissues acquired in the course of clinical care and treatment. This contrasts with other recent papers that focus on optimal fields of view. Second, it shows that it is often possible to increase segmentation accuracy by including additional data (NES) on nuclear envelop morphology, and it proposes a broadly useful antibody cocktail. Third, and most significantly, it shows that the addition of real augmentations comprising defocused and saturated images to model training data improves segmentation accuracy to a significant extent whereas augmentations based on Gaussian blurring provide substantially less benefit. These results extend to deep learning frameworks based on instance segmentation (UnMICST-M) and on semantic segmentation (UnMICST-U and UnMICST-P). Finally, using newly generated labeled training data for multiple tissue types, it shows that real augmentation and NES combine to improve the robustness and accuracy of segmentation across many tissues; these improvements are directly applicable to the real-world task of segmenting high dimensional tissue and tumor images. The magnitude of improvement observed by the inclusion of NES data or real augmentation is substantially greater than the differences observed between ML frameworks. UnMICST models, therefore, represent a good starting point for performing image segmentation on rapidly growing tissue data repositories. Errors remaining when multiplexed images are segmented using optimized UnMICST models appear to have a subtle biological basis. The development of additional physiology-aware machine-learning models may be necessary to reduce these apparent errors.

One of the surprises in the current work was the seemingly low level of agreement achieved by two human experts annotating the same image data; we estimated that only 60% of the annotated nuclei between annotators had an overlap of 60% or greater (0.6 IoU threshold). Poor agreement is almost certainly a consequence of our use of a stringent sweeping IoU scoring criterion that measures the fraction of pixels that overlap between two segmentation masks. The alternative, and widely-used F1 score, which determines whether two observers (or an observer and a machine) agree on the presence of a nucleus, achieves inter-observer and automated segmentation accuracy of 0.78, which is comparable to the highest F1-scoring tissue reported for Mesmer^40^, another deep learning model applied to tissue images. Moreover, our results with IoU values are similar to those recently reported by Kromp et al.^17^ (when IoU thresholds are adjusted to enable direct comparison). The authors of Cellseg^41^ also report comparable segmentation accuracies and note the difficulty of achieving a high IoU value with cells that vary dramatically in shape and focus.

It would therefore appear that many studies have achieved similar levels of inter-observer agreement and that our results are not an outlier, even though we include problematic data. This points to a fundamental challenge for all supervised learning approaches whose solution is not immediately clear. Collection of precise 3D data followed by the imposition of different levels of blurring and addition of intensity artefacts will be needed to understand the origins of inter-observer disagreement in tissue images and achieve higher quality training and test data. It also seems likely that practical improvements in segmentation are likely to come from combining recently described advances. For example, Greenwald et al.^40^ use a clever community-based approach to acquire much more training data than in the current work, Kromp et al.^17^ combine tissue images with ground truth annotation acquired from cultured cells (by a team of undergraduate students), whereas the current work focuses on the use of NES and real augmentations to improve the robustness of segmentation algorithms across the board.

From a machine learning perspective, the value of adding additional image channels to training data is self-evident. Experimental feasibility is not always so clear. A key tradeoff is that the greater the number of fluorescence channels used for segmentation, the fewer the channels available for the collection of data on other markers. Fortunately, the development of highly multiplexed imaging has made this less relevant because collection of 20–40 or more image channels (each corresponding to a different fluorescent antibody) has become routine. This makes it straightforward to reserve two channels for segmentation. The cost-benefit ratio of adding extra segmentation data will be different in high content screening of cells in multi-well plates, for which inexpensive reagents are generally essential than in tissue imaging. In tissues, the morphology of nuclear lamin changes with disease state^42^, cell type, activation state and numerous other biological processes. While these challenges segmentation routines, imaging lamins is also likely to provide valuable biological information, further arguing for routine collection of these data^43^. To allow others to build on the current work, we are releasing all training and test images, their segmentation masks and annotations, and real augmentations for multiple types of tissue (tonsil, ovary, small intestine and cancers of the colon, brain, lung, prostate) via the EMIT resource; models are released as components of the UnMICST model resource (see data availability and code availability information).

The most immediately generalizable finding from this work is that real augmentation outperforms computed augmentation generated using Gaussian kernels. Blurring and image saturation are an inevitable consequence of the limited bandwidth of optical systems, the thickness of specimens relative to the depth of field, light scattering, diffraction, the use of non-immersion objective lenses and consequent refractive index mismatches, and a variety of other physical processes. Real out-of-focus blur also differs when the focal plane is above and below the specimen. Areas for future application of real augmentations could include inhomogeneous light sources and stage jitter. It will undoubtedly be useful to determine kernels for more effective computed augmentation, but collecting real augmentation data imposes a minimal burden in a real-world setting. Our observation that real augmentation outperforms computed augmentation may also have general significance outside of the field of microscopy: with any high-performance camera system, real out-of-focus data will inevitably be more complicated than Gaussian blur.

## Methods

### Sample preparation for imaging

To generate images for model training and testing, human tissue specimens from multiple patients were used to construct a multi-tissue microarray (HTMA427) under an excess (discarded) tissue protocol approved by the Institutional Review Board (IRB) at Brigham and Women’s Hospital (BWH IRB 2018P001627). One or two 1.5 mm diameter cores were taken from tissue regions with the goal of acquiring one or two examples of different healthy or tumor types including non-neoplastic medical diseases and secondary lymphoid tissues such as tonsil. Slides were stained with reagents from Cell Signaling Technologies (Beverly MA, USA) and Abcam (Cambridge UK) as shown in Table 1.

**Table 1:** Antibodies used for immunofluorescence staining.

Target	Fluorochrome	Species	Clone	Vendor	Cat. No.	RRID
DNA	Hoechst 33342	NA	NA	CST	4082	AB_10626776
Lamin B2	Alexafluor 647	Rabbit	EPR9701(B)	Abcam	ab200427	AB_2889288
NUP98	Alexafluor 647	Rabbit	C39A3	CST	13393	AB_2728831
Lamin B1	Alexafluor 488	Rabbit	EPR8985(B)	Abcam	ab194106	AB_2728786
Lamin A/C	Alexafluor 488	Mouse	4C11	CST	8617S	AB_10997529
Lamin B receptor	Alexafluor 488	Rabbit	E398L	Abcam	ab201532	AB_2889290

Before imaging, slides were mounted with 90% glycerol and a #1.5 coverslip. Prior to algorithmic evaluation, the images were split into three mutually disjoint subsets and used for training, validation, and testing.

#### Acquisition of image data and real augmentations

The stained TMA was imaged on a INCell 6000 (General Electric Life Sciences) microscope equipped with a 20×/0.75 objective lens (370 nm nominal lateral resolution at 550 nm wavelength) and a pixel size of 0.325 µm per pixel. Hoechst and lamin-A647 were excited with a 405 and 642 nm laser, respectively. Emission was collected with the DAPI (455/50 nm) and Cy5 (682/60 nm) filter sets with exposure times of 60 and 100 ms, respectively. Whole-slide imaging involved acquisition of 1215 tiles with an 8% overlap, which is recommended for stitching in ASHLAR, a next generation stitching and registration algorithm for large images (https://github.com/labsyspharm/ashlar). To generate defocused data, we acquired images from above and below the focal plane by varying the *Z*-axis by 3 µm in both directions. To generate saturated images of DNA staining, a 150 ms exposure time was used. These two types of suboptimal data were then used for real augmentation during model training, as described below.

Representative cores for lung adenocarcinoma, non-neoplastic small intestine, normal prostate, colon adenocarcinoma, glioblastoma, non-neoplastic ovary, and tonsil were extracted from image mosaics and down-sampled by a factor of 2 to match the pixel size of images routinely acquired and analyzed in MCMICRO^31^. Images were then cropped to 256 × 256-pixel tiles, and in-focus DNA and NES were imported into Adobe Photoshop to facilitate human annotation of nuclear boundaries. We labeled contours and background classes on separate layers while swapping between DNA and NES as necessary. To save time, we drew complete contours of nuclei and filled these in using the Matlab *imfill* operation to generate nuclei centers. For nuclei at the image borders where contours would be incomplete, we manually annotated nuclei centers. As described by Ronneberger et al. (2015), a fourth layer was used to mark areas between clumped cells. These additional annotations made it possible to specifically penalize models that incorrectly classified these pixels. During image review, we observed that certain nuclei morphologies appeared more frequently than others. To account for this imbalance, we annotated only characteristic nuclei of each tissue type in each image in an effort to balance the occurrence of nuclei shapes in our training, validation, and test sets. For example, small intestine and colon images displayed both round and elongated nuclei, and since the former shape was already present in other tissues (such as lung) in our dataset, we only annotated the latter shape for small intestine and colon tissues. Full dense annotations on a held-out test dataset were validated by a second annotator and measured using the F1-score. The F1-score evaluation between both annotated ground truths was high and demonstrated excellent agreement (Supplementary Information 1).

Because original, defocused, and saturated images of DNA were all acquired in the same image stack, it was possible to use a single registered set of DNA annotations across all augmented image channels. To produce the training set, each image was cropped into 64 × 64 patches, normalized to use the full dynamic range, and further augmented using 90° rotations, reflections, and 20% upscaling. Consistent with the training set, the validation and test sets also include defocused and saturated examples but were not augmented with standard transformations. The ratio of data examples present in the training, validation, and test set split was 0.36:0.24:0.4. For a fair comparison across models, the same dataset and split were used for the three deep learning frameworks described in this manuscript (Supplementary Table 2).

#### Model implementation

To facilitate model training, three distinct state-of-the-art architectures were separately, trained, implemented, and evaluated. They are, in no particular order, UNet, Mask R-CNN, and PSPNet and were adopted from their original references without modification to their architecture. UNet was selected for its prior success in the biomedical domain, Mask R-CNN was selected for its ability to perform both object detection and mask generation, and PSPNet was selected for its capacity to integrate image features from multiple spatial scales. Training, validation, and test data were derived from 12 cores in 7 tissues and a total of 10,359 nuclei in the composition of colon – 1142; glioblastoma (GBM) – 675; lung – 1735; ovarian – 956; fibroblast – 922; small intestine – 1677; tonsil – 3252. To maintain consistency of evaluation across segmentation algorithms, segmentation accuracy was calculated by counting the fraction of cells in a held out test set that passed a sweeping Intersection over Union (IoU) threshold. The NES channel was concatenated to the DNA channel as a three-dimensional array as input into each architecture.

#### UnMICST-U model training

A three-class UNet model^14^ was trained based on annotation of nuclei centers, nuclei contours, and background. The neural network is comprised of 4 layers and 80 input features. Training was performed using a batch size of 32 with the Adam Optimizer and a learning rate of 0.00005 with a decay rate of 0.98 every 5000 steps until there was no improvement in accuracy or ~100 epochs had been reached. Batch normalization was used to improve training speed. During training, the bottom layer had a dropout rate of 0.35, and L1 regularization was implemented to minimize overfitting^44,45^ and early stopping. Training was performed on workstations equipped with NVidia GTX 1080 or NVidia TitanX GPUs.

#### UnMICST-M model training

Many segmentation models are based on the Mask R-CNN architecture^15^, Mask R-CNN has previously exhibited excellent performance on a variety of segmentation tasks. Mask R-CNN begins by detecting bounding boxes of nuclei and subsequently performs segmentation within each box. This approach eliminates the need for an intermediate watershed, or equivalent, segmentation step. Thus, Mask R-CNN directly calculates a segmentation mask, significantly reducing the overhead in traditional segmentation pipelines. We adopted a ResNet50^46^ backbone model in the UnMICST-M implementation and initialized the weights using pretrained values from the COCO object instance segmentation challenge^33^ to improve convergence properties. For efficient training, we upsampled the original input images to 800 × 800-pixels and trained a model for 24 epochs using a batch size of 8. The Adam optimizer, with a weight decay of 0.0001 to prevent overfitting, was exploited with a variable learning rate, initially set to 0.01 and decreased by a factor of 0.1 at epochs 16 and 22. Training was performed on a compute node cluster using 4 NVidia TitanX or NVidia Tesla V100 GPUs. For evaluation and comparison, we used the model with the highest performance on the validation set, following standard practice.

#### UnMICST-P model training

We trained a three class PSPNet model^47^ to extract cell nuclei centers, nuclei contours, and background from a wide variety of tissue types. PSPNet is one of the most widely used CNNs for the semantic segmentation of natural scene images in the computer vision field. The network employs a so-called pyramid pooling module whose purpose is to learn global as well as local features. The additional contextual information used by PSPNet allowed the segmentation algorithm to produce realistic probability maps with greater confidence. We used ResNet101 as a backbone. Training of the network was performed using a batch size of 8 with an image size of 256 × 256-pixels for 15,000 iterations or until the minimum loss model was obtained. A standard cross entropy loss function was used during training. Gradient descent was performed using the Adam optimizer with a learning rate of 0.0001 and a weight decay parameter of 0.005 via L2 regularization. Batch normalization was employed for faster convergence, and a dropout probability of 0.5 was used in the final network layer to mitigate overfitting. The model training was performed on a compute cluster node equipped with NVidia Tesla V100 GPUs.

#### Analysis of multi-dimensional data

For the analysis shown in Fig. 6, a 64-plex CyCIF image of non-neoplastic small intestine tissue from the EMIT TMA (https://www.synapse.org/#!Synapse:syn22345748/) was stained with a total of 45 antibodies as described in protocols https://www.protocols.io/view/ffpe-tissue-pre-treatment-before-t-cycif-on-leica-bji2kkge and 10.17504/protocols.io.bjiukkew. Images were segmented using the UnMICST-U model trained on DNA with NES data and real augmentations. Mean fluorescence intensities across 45 markers for 27,847 segmented nuclei were quantified as described in ref. ^31^. E-cadherin positive and CD45 positive cells were identified using Gaussian-mixture models on log-transformed data. For multivariate clustering, log-transformed mean intensities of all single cells of 14 selected protein markers (E-cadherin, pan-cytokeratin, CD45 CD4, CD3D, CD8, RF3, PML, GLUT1, GAPDH TDP43, OGT, COLL4, an EPCAM) were pre-processed using Uniform Manifold Approximation and Projection (UMAP)^48^ and clustered using Hierarchical Density-Based Spatial Clustering of Applications with Noise (HDBSCAN)^49^. Clusters expressing a high level of both E-cadherin and CD45 were identified and overlaid on a false-colored image showing the staining of DNA, E-cadherin, and CD45.

## Supplementary information

Supplementary Information

Description of Additional Supplementary Data

supplementary data

nr-reporting-summary

## Data Availability

To allow others to build on the current work, we are releasing all training, validation and test images, their annotations, and real augmentations for multiple types of tissue (tonsil, ovary, small intestine and cancers of the colon, brain, lung, prostate) via the EMIT resource; models for training and inference are released as components of the UnMICST model resource. Source data for graphs in main figures can be found in Supplementary Data 1.xlsx.

## Code availability

The code and instructions used for training and implementing the UnMICST models can be found at: https://labsyspharm.github.io/UnMICST-info/
