# Supplementary material for: UnMICST: Deep learning with real augmentation for robust segmentation of highly multiplexed images of human tissues
Source: Commun Biol. 2022 Nov 18;5:1263. doi: 10.1038/s42003-022-04076-3 (PMC9674686; doi:10.1038/s42003-022-04076-3)
Supplement: Supplementary file 4 — nr-reporting-summary [file 42003_2022_4076_MOESM4_ESM.pdf]

nature portfolio

Corresponding author(s): Peter Sorger

Last updated by author(s): Aug 6, 2022

Reporting Summary

Nature Portfolio wishes to improve the reproducibility of the work that we publish. This form provides structure for consistency and transparency in reporting. For further information on Nature Portfolio policies, see our Editorial Policies and the Editorial Policy Checklist.

Statistics

For all statistical analyses, confirm that the following items are present in the figure legend, table legend, main text, or Methods section.

n/a	Confirmed	
☐	☒	The exact sample size (*n*) for each experimental group/condition, given as a discrete number and unit of measurement
☒	☐	A statement on whether measurements were taken from distinct samples or whether the same sample was measured repeatedly
☒	☐	The statistical test(s) used AND whether they are one- or two-sided<br>*Only common tests should be described solely by name; describe more complex techniques in the Methods section.*
☒	☐	A description of all covariates tested
☒	☐	A description of any assumptions or corrections, such as tests of normality and adjustment for multiple comparisons
☒	☐	A full description of the statistical parameters including central tendency (e.g. means) or other basic estimates (e.g. regression coefficient) AND variation (e.g. standard deviation) or associated estimates of uncertainty (e.g. confidence intervals)
☒	☐	For null hypothesis testing, the test statistic (e.g. *F*, *t*, *r*) with confidence intervals, effect sizes, degrees of freedom and *P* value noted<br>*Give P values as exact values whenever suitable.*
☒	☐	For Bayesian analysis, information on the choice of priors and Markov chain Monte Carlo settings
☒	☐	For hierarchical and complex designs, identification of the appropriate level for tests and full reporting of outcomes
☒	☐	Estimates of effect sizes (e.g. Cohen's *d*, Pearson's *r*), indicating how they were calculated

Our web collection on statistics for biologists contains articles on many of the points above.

Software and code

Policy information about availability of computer code

Data collection	Matlab 2018b, Tensorflow 1.14, Python 3.6, PyTorch, Photoshop 2021
Data analysis	Intersection-over-union script in Matlab 2018b

For manuscripts utilizing custom algorithms or software that are central to the research but not yet described in published literature, software must be made available to editors and reviewers. We strongly encourage code deposition in a community repository (e.g. GitHub). See the Nature Portfolio guidelines for submitting code & software for further information.

Data

Policy information about availability of data

All manuscripts must include a data availability statement. This statement should provide the following information, where applicable:

- Accession codes, unique identifiers, or web links for publicly available datasets
- A description of any restrictions on data availability
- For clinical datasets or third party data, please ensure that the statement adheres to our policy

UnMICST unique identifier is RRID:SCR_021050.
Data and models are available for download: https://www.synapse.org/#!Synapse:syn24192218/files/
Description can be found here: https://labsyspharm.github.io/UnMICST-info/
MIT license.

Human research participants

Policy information about studies involving human research participants and Sex and Gender in Research.

Reporting on sex and gender	Sex and gender information not collected because they are not relevant to this study.
Population characteristics	Human and animal subjects not included in this study.
Recruitment	No participants were recruited.
Ethics oversight	Human and animal subjects not included in this study.

Note that full information on the approval of the study protocol must also be provided in the manuscript.

Field-specific reporting

Please select the one below that is the best fit for your research. If you are not sure, read the appropriate sections before making your selection.

☒ Life sciences ☐ Behavioural & social sciences ☐ Ecological, evolutionary & environmental sciences

For a reference copy of the document with all sections, see nature.com/documents/nr-reporting-summary-flat.pdf

Life sciences study design

All studies must disclose on these points even when the disclosure is negative.

Sample size	Sample sizes depended on availability of manual annotations. The annotations were derived from available clinical discard tissue specimens. The selection of the tissue types was based on their morphologically distinctness.
Data exclusions	No data was excluded.
Replication	Conclusions were successfully reproduced on 3 different deep learning model architectures.
Randomization	Data was randomly assigned to training, validation, and test sets.
Blinding	Blinding not relevant to developing image analysis deep learning models. Models were objectively assessed using common metrics.

Reporting for specific materials, systems and methods

We require information from authors about some types of materials, experimental systems and methods used in many studies. Here, indicate whether each material, system or method listed is relevant to your study. If you are not sure if a list item applies to your research, read the appropriate section before selecting a response.

Materials & experimental systems

n/a	Involved in the study	
☐	☒	Antibodies
☒	☐	Eukaryotic cell lines
☒	☐	Palaeontology and archaeology
☒	☐	Animals and other organisms
☒	☐	Clinical data
☒	☐	Dual use research of concern

Methods

n/a	Involved in the study	
☒	☐	ChIP-seq
☒	☐	Flow cytometry
☒	☐	MRI-based neuroimaging

Antibodies

Antibodies used

Abcam ab200427; EPR9701(B)
CST 13393 ; C39A3
Abcam ab194106; EPR8985(B)
CST 8617S; 4C11
Abcam ab201532; E398L

Validation

Antibodies were selected from cycif.org, which were tested on multiple tissue specimens including positive and negative controls. Conjugated antibodies were tested at different concentrations to optimize signal-to-noise ratio. Procedure can be found in PMCID: PMC6959005.
